# Sequential Steps of Chromosomal Differentiation in Atlantic Surgeonfishes: Evolutionary Inferences

**DOI:** 10.1155/2014/825703

**Published:** 2014-08-12

**Authors:** Paulo Roberto Antunes de Mello Affonso, Maria Aparecida Fernandes, Josivanda Santos Almeida, Wagner Franco Molina

**Affiliations:** ^1^Departamento de Ciências Biológicas, Universidade Estadual do Sudoeste da Bahia, 45206-150 Jequié, BA, Brazil; ^2^Departamento de Biologica Celular e Genética, Universidade Federal do Rio Grande do Norte, 59078-970 Natal, RN, Brazil

## Abstract

Surgeonfishes are a species-rich group and a major biomass on coral reefs. Three species are commonly found throughout South Atlantic, *Acanthurus bahianus*, *A. chirurgus*, and *A. coeruleus*. In this paper, we present the first cytogenetic data of these species, revealing a sequential chromosomal diversification. *A. coeruleus* was characterized by a relatively conserved karyotype evolved by pericentric inversions of some pairs (2*n* = 48, 2sm + 4st + 42a). In contrast, the karyotypes of *A. bahianus* (2*n* = 36) and *A. chirurgus* (2*n* = 34) were highly differentiated by the presence of six large metacentric pairs in *A. bahianus* (12m + 2sm + 4st + 18a) and *A. chirurgus* (12m + 2sm + 4st +1 6a) probably derived by chromosomal fusions that corroborate their closer relationship. A discernible *in tandem* fusion represents an autapomorphic character to *A. chirurgus*. In spite of macrostructure variation, single nucleolar organizer regions (NORs) on short arms of a subtelocentric pair and similar distribution of C-bands were observed in the three species. Overlapping of chromosomal data with molecular phylogeny indicated pericentric inversions which took place nearly at 19 Ma while centric fusions are as recent as 5 Ma. A physical mapping of coding and noncoding sequences in *Acanthurus* could clarify the role of additional rearrangements during their chromosomal evolution.

## 1. Introduction

Acanthuridae are a monophyletic fish family composed of about 80 species, popularly known as surgeonfishes or tangs [1]. This is an ancient group (nearly 54 Ma) and most of genera (*Acanthurus*,* Naso*,* Paracanthurus*,* Zebrasoma,* and* Ctenochaetus*) diverged between 17 and 21 Ma in Early Miocene [2].

Apparently, the Pacific Ocean is the center of origin of surgeonfishes, retaining most of Acanthuridae richness [3, 4]. Further colonization events resulted in distribution of this family to virtually all tropical and subtropical seas of the world, but Mediterranean Sea [1, 3]. The genus* Acanthurus* is the largest within the family, but monophyly of the genus is still controversial [2–5]. This fish group is morphologically and ecologically diversified, mainly in relation to foraging behavior and dentition, composing one of the most representative herbivorous fish group on coral reefs [2, 6].

A total of four species of Acanthuridae, all belonging to the genus* Acanthurus*, are present in western Atlantic [7]. Three species are common along the Brazilian coast (Western South Atlantic):* Acanthurus coeruleus *(blue tang),* A. bahianus *(barber surgeonfish), and* A. chirurgus* (doctorfish) [7, 8]. Another* Acanthurus* species (*A. monroviae*) was also recorded off southeastern coast of Brazil, but it seems to be an occasional occurrence [9]. Moreover,* A. bahianus* was thought to range from USA to southern Brazil, but morphological and genetic analyses have shown that populations from Massachusetts to Caribbean actually refer to another species, validated as* A. tractus* [7].

In spite of the low diversity of Atlantic species when compared to Pacific and Indian oceans, surgeonfishes are a dominant fish group forming large assemblages in several reef areas from South Atlantic [3].* A. coeruleus* specimens are usually solitary due to their territoriality behavior, while* A*.* bahianus* and* A*.* chirurgus* are commonly found in small to large schools, depending on the ontogenetic stage [10]. As most acanthurids, these species present relatively long pelagic larval stages with a mean duration from 51.6 to 55.2 days [11]. Usually, wide-range reef fish species with long pelagic larval development are characterized by a lack of genetic subdivision among populations [12] and low rates of chromosomal variation [13].

However, reports about cytogenetic patterns of Acanthuridae are still underrepresented (less than 5% of species) and restricted to Indo-Pacific species [14]. The three analyzed species from Pacific,* Acanthurus triostegus*,* Prionurus scalprum* [15], and* Ctenochaetus striatus* [16], all share a conservative Perciformes-like karyotype with 2*n* = 48a, considered basal to this fish group [17].

In order to increase the karyotypic data of Acanthuridae and to infer the chromosomal evolution of Atlantic species, cytogenetical analyses were carried out for three Acanthuridae species from Brazilian coast, South Atlantic.

## 2. Material and Methods

**Figure 1:**
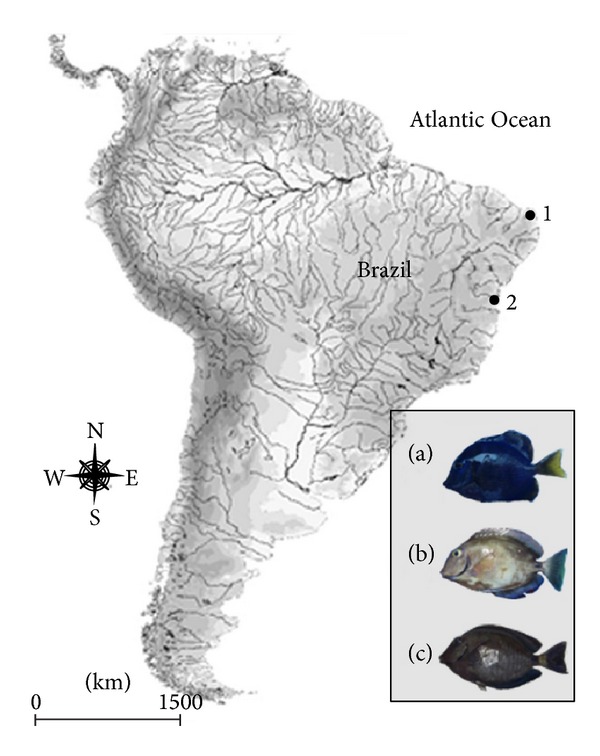
Map of South America showing the collections sites of* Acanthurus coeruleus* (a),* A. bahianus* (b), and* A. chirurgus* (c) in the states of Rio Grande do Norte (1) and Bahia (2), northeastern Brazil.

Nine individuals of* Acanthurus coeruleus*, four individuals of* A. bahianus*, and 17 individuals of* A. chirurgus* were cytogenetically studied. Animals were collected using hand nets (60 × 100 cm) by snorkeling at coastal reef areas from the states of Rio Grande do Norte (5°46′S, 35°12′W) and Bahia (13°00′S, 38°32′W and 13°52′S, 38°56′W) in northeastern Brazilian shore (Figure 1). Right after collection, specimens were transported in plastic bags with oxygen to the laboratories and placed in 60 L tanks equipped with filtration and aeration systems.

Twenty-four hours prior to chromosomal preparation, the animals were inoculated via intramuscular with a solution of antigen complexes (Munolan) for mitotic induction [18]. After this period, the specimens were anesthetized and euthanized by immersion in in water at 0–4°C up to complete interruption of gill movements [19]. To obtain mitotic chromosomes* in vitro*, portions of anterior kidney were removed and transferred to RPMI medium (Cultilab) with about 50 *μ*L of 0.025% colchicine, followed by hypotonic treatment (KCL 0.075 M) for 20 minutes at 37°C and fixation in Carnoy's fixative (methanol : acetic acid 3 : 1) [20]. Chromosomes were stained with 5% Giemsa in phosphate buffer (pH 6.8) for karyotypic analyses. Nucleolus organizer regions (NORs) were detected by silver nitrate staining (Ag-NORs) [21], whereas heterochromatic regions were evidenced by C-banding [22].

Metaphases were photographed using an Olympus BX51 (Olympus, Tokyo, Japan) epifluorescence photomicroscope equipped with digital capture system. Chromosomes were classified as metacentric (m), submetacentric (sm), subtelocentric (st), and acrocentric (a) based on arm ratio [23]. The pairs were arranged in decreasing order size according to each morphological category (m, sm, st, and a) in karyotypes using the software Adobe Photoshop CS6 v. 13.0.

## 3. Results

**Figure 2:**
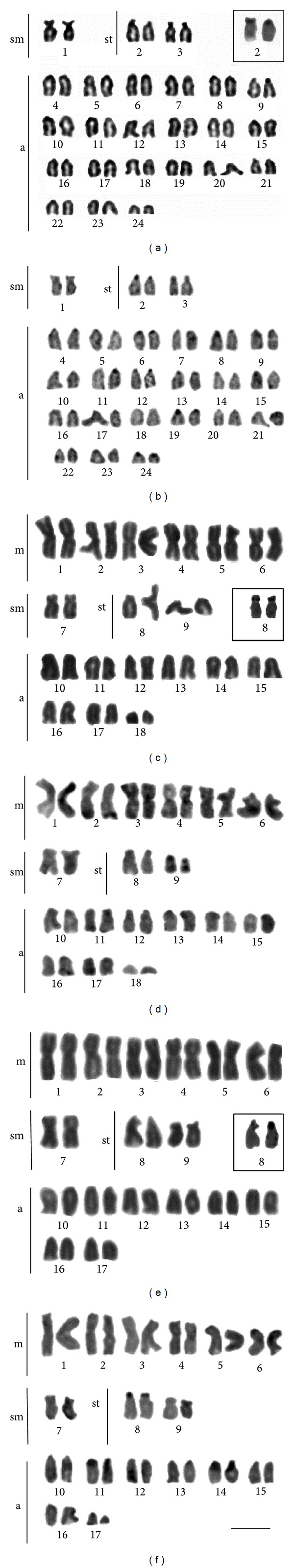
Karyotypes of* Acanthurus coeruleus* ((a) and (b)) with 2*n* = 48,* A. bahianus* ((c) and (d)) with 2*n* = 36, and* A. chirurgus* ((e) and (f)) with 2*n* = 34 after conventional Giemsa staining ((a), (c), and (e)) and C-banding ((b), (d), and (f)). The NOR-bearing chromosomes after silver nitrate staining of each species are shown in boxes (pair 2 of* A. coeruleus* and pair 8 of* A. bahianus* and* A. chirurgus*).

The three* Acanthurus* species showed remarkable karyotype diversification.* Acanthurus coeruleus* presented 2*n* = 48, composed of two submetacentric, four subtelocentric, and 42 acrocentric chromosomes (Figure 2(a)). The diploid number of* A. bahianus* equals 2*n* = 36 with a karyotype composed of 12 large metacentric, two submetacentric, four subtelocentric, and 18 acrocentric chromosomes (Figure 2(c)) while* A. chirurgus* was characterized by 12 large metacentric, two submetacentric, four subtelocentric, and 16 acrocentric chromosomes (2*n* = 34) (Figure 2(e)).

Small amounts of heterochromatin were detected mainly at pericentromeric regions and interspersed with NORs in studied species (Figures 2(b), 2(d), and 2(f)). In* A. bahianus*, terminal C-bands were also observed in some pairs (Figure 2(d)).

Single nucleolar organizer regions (NORs) were located by silver nitrate staining on short arms of the largest subtelocentric pair in the three Acanthuridae species (Figure 2, inbox).

**Figure 3:**
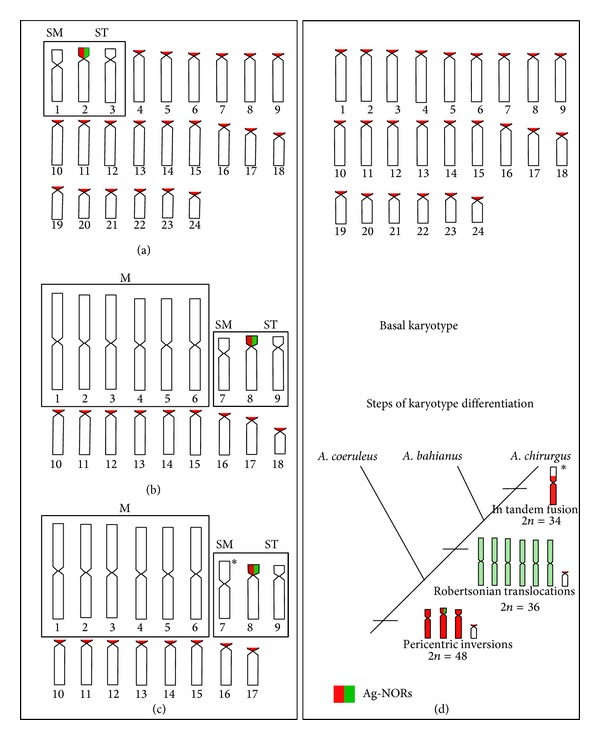
Idiograms of chromosomes sets of* Acanthurus coeruleus *(a),* A. bahianus *(b), and* A. chirurgus *(c) showing the most conspicuous shared cytogenetic traits in boxes. In (d), the basal karyotype of Perciformes (2*n* = 48a) and a phylogenetic hypothesis based on sequential chromosomal rearrangements inferred in the three* Acanthurus *species.

Based on chromosomal data, idiograms were generated to highlight particular karyotype traits for each species and the inferred pathways of chromosomal differentiation based on a phylogenetic hypothesis to Atlantic* Acanthurus* (Figures 3(a)–3(d)).

## 4. Discussion

It is assumed that the presence of 48 acrocentric chromosomes represents a plesiomorphic feature within Perciformes [17, 24]. This condition is particularly frequent among marine fish and could be related to dispersal abilities (high gene flow) between populations, thereby preventing the fixation of new chromosomal rearrangements and karyotypic divergence [13]. In fact, the low genetic structure in reef fish species has been correlated to the production of planktonic eggs and/or larvae that can be dispersed over large distances [12].

This trend (2*n* = 48a) seems to be valid for Acanthuridae species from Indo-Pacific Ocean of different genera, such as* Acanthurus*,* Ctenochaetus*, and* Prionurus* [14]. However, inconsistent relationship between pelagic larval duration (PLD) and genetic connectivity or chromosomal patterns has been reported in some marine species [11, 25]. In these cases, ecological and biogeographic aspects of each species might be more relevant to explain the genetic variation than PLD itself, as observed in the present study.

As expected for widely distributed species with long PLD,* A. coeruleus* presented typical Perciformes-like features, that is, a diploid number of 48, single NORs, and a large number of acrocentric chromosomes (Figure 2(a)). The karyotype of this species (2sm + 4st + 42a) demonstrates the occurrence of pericentric inversions in three chromosome pairs (1st, 2nd, and 3rd pairs), a common rearrangement in Perciformes that accounts for most of karyotype diversification in marine fish [24]. A similar set of three chromosomal pairs in both morphology and size is also observed in* A. bahianus* and* A. chirurgus*, represented by a submetacentric pair and two subtelocentric pairs, including the NOR-bearing pair. Because of the high resemblance of such pairs in the three* Acanthurus* species, they are supposed to share a common origin before the differentiation of each lineage, thereby indicating a symplesiomorphic trait. Estimates of divergence time between the subclade that comprises* A. coeruleus* and that clusters* A. chirurgus* and* A. tractus* [2], a sister-species of* A. bahianus*, suggest that these putative homeologous pairs (sm and st) had arisen at nearly 19 Ma.

On the other hand,* A*.* bahianus* and* A. chirurgus* presented an evolutionary chromosomal pattern rarely found in typical marine fishes. The drastic reduction in diploid number from 48 chromosomes to 2*n* = 36 and 2*n* = 34, respectively, along the presence of large metacentric pairs is evidence of sequential Robertsonian rearrangements or centric fusions (Figures 2(c) and 2(e)). Indeed, the uniqueness of Robertsonian translocations in karyotypes of* A. bahianus* and* A. chirurgus* (Figure 3), and similar size of metacentric pairs reinforces these rearrangements are a recently shared trait between both species. An additional fusion representing an autapomorphic condition is presented in* A. chirurgus* karyotype since this species has an exclusive large sm pair (7th) and lacks the smallest acrocentric pair observed in the other two species (Figure 2(e)).

The chromosomal speciation observed in* Acanthurus* species of South Atlantic is likely to reflect historical events. Extensive analysis of biogeography and evolution of reef fish from Atlantic indicated that changes in ocean dynamics over the past 10 Ma have determined the differential richness and endemism levels of fish genera and families of reef fish [26]. As discussed by Galetti et al. [24], the rate of chromosomal evolution in reef fish from Atlantic Ocean also seems to be strongly related to habitat isolation of coastal areas during glaciation periods followed by further sea level uprising.

Unfortunately, no reports about time of divergence between* A. bahianus* and* A. chirurgus* are available, thus hindering the minimum time span after Robertsonian rearrangements that gave rise to the large metacentric chromosomes, herein referred as a single trait. However, estimates inferred for* A. chirurgus* and* A. tractus* [2], being the latter a sibling species of* A. bahianus* [7], point out that these rearrangements took place by at least 5 Ma. Even though the time estimates for the occurrence of chromosomal fusions in both* Acanthurus* species might require some bias correction, they are intermediary to periods of major biogeographic isolation events in Atlantic Ocean such as Amazon outflow (~10 Ma) and uplifting of Panama isthmus (~3 Ma) [27]. Nonetheless, the influence of these biogeographic barriers in the putative fixation of chromosomal rearrangements remains unclear and further cytogenetic studies in other species, particularly* A. tractus*, are highly encouraged.

Different from pericentric inversions, centric fusions or Robertsonian rearrangements are usually reported in non-Perciformes marine fish, such as flatfish (Pleuronectiformes) [28], toadfish (Batrachoidiformes) [29], and some mullets (Mugiliformes) [30]. Centric fusions are particularly common in Batoidea (stingrays, guitarfish, and skates), the most derived superorder of elasmobranchs [31]. Conversely, these rearrangements have been scarcely identified in Perciformes at a polymorphic stage in Pomacentridae [32], Gobiidae [33, 34], Lutjanidae [35], Apogonidae [36, 37], and Uranoscopidae [38] or else restricted to a particular taxon like* Sparisoma* (parrotfishes) [39]. Therefore, the karyotypes of* A. bahianus* and* A. chirurgus* can be regarded as highly derived in relation to basal karyotype suggested to Perciformes.

While the macrostructure was variable, the NOR-bearing chromosomes seem to be conserved in the three species (Figure 2, inbox). This pattern indicates that these chromosomal regions as poor cytotaxonomic markers, differing from other Atlantic fishes in which the identification of ribosomal cistrons has proved to be efficient to distinguish apparently homogeneous karyotypes, as in Lutjanidae [40], Serranidae [41], and Gerreidae [42] or even population units [43, 44]. Similarly, heterochromatin was virtually similar among* A. coeruleus*,* A. bahianus*, and* A. chirurgus*, being mainly dispersed over centromeres and NORs, as commonly found in most Perciformes [45]. Therefore, it is unclear if the deep divergences in these fish karyotypes are followed by microstructural changes. Further analyses using other banding methodologies and mapping of sequences by fluorescence* in situ* hybridization (FISH) are required to evaluate the extension of such apparent homogeneity of specific chromosomal regions in* Acanthurus*.

The amount of chromosomal traits in the three* Acanthurus* species from Brazilian coast allows raising a phylogenetic hypothesis to them. Indeed, the ordination and sharing of the traits show a closer phylogenetic relationship between* A. bahianus* and* A. chirurgus* than to* A. coeruleus*. This result is corroborated by previous genetic analyses. Indeed, analysis of* CytB* sequences showed a more basal condition between* A. coeruleus* in relation to the other two congeners [7]. Recently, a phylogenetic analysis of Acanthuridae based on sequence data of two mitochondrial and seven nuclear genes [2] corroborated the ancestral position of* A. coeruleus* in relation to* A. chirurgus* and* A. tractus*, which replaces* A. bahianus* in the Caribbean [7, 46].

Thus, for analyzed Atlantic species, the chromosomal traits show robust support to clarify the phylogenetic arrangement among them serving as useful markers to evolutionary studies in Acanthuridae. Moreover, in agreement with phylogeographic studies [11, 26], the chromosomal differences between Atlantic species of* Acanthurus* seem to be more related to ecology and evolutionary history than to dispersal potential since the three species share a relatively long PLD.

## 5. Conclusion

In conclusion, the chromosomal analyses in* Acanthurus* allowed identifying sequential events related to speciation process that differ from most cytogenetical reports on marine Perciformes, where specific rearrangements are often unclear. A step-by-step karyotype modification can be inferred from the most basal pattern, involving few structural rearrangements (pericentric inversions in* A. coeruleus*) to high derived ones, originated by Robertsonian fusions in both* A. bahianus* and* A. chirurgus* and additional in tandem fusion in* A. chirurgus*. This scenario reveals a unique condition to tracing back the order of chromosomal evolutionary changes in Atlantic surgeonfish.
